# The Bristol Royal Infirmary Impasse

**Published:** 1907-10-12

**Authors:** P. Watson Williams


### THE BRISTOL ROYAL INFIRMARY IMPASSE.

By P. WATSON WILLIAMS, M.D.

As we have already referred at some considerable length to the conflict of opinion at Bristol between the lay Committee of Governors of the Royal Infirmary and the Faculty of Medicine, concerning the new rules and regulations which the former desires to impose upon the Honorary Staff of the Infirmary, we have pleasure in giving equal prominence to the following communication.

Dr. Watson Williams writes (October 7):—I think it is incumbent on me to offer an explanation and correction on a point touched on in your editorial with reference to the Bristol Royal Infirmary, on page 19 in your issue October 5, as a matter of simple courtesy due to the Committee at which I had to represent the views of my colleagues. You say the President stated that I, as "representative of the Faculty, acquiesced in the proposed new rules, if I could not get anything more." It was unfortunate that I was unable to be present at the Board meeting, in your report of which these remarks appeared, as I was ill at the time. At the commencement of your article you give verbatim the resolution of the Committee. But the rules had come up annotated by the Faculty, and contained further wording to the effect that the Faculty considered the rule as it stood infeasible, and that they *assumed* certain alterations were contemplated. It was also accompanied by a statement that the Faculty did not recede from the position adopted in their letter to the President in April last—namely, that, having given their services free to one hospital, and having spent much time in doing their work to the best of their ability at that institution, as a matter of principle it is only just that they should be free to use their spare time as they thought best.

Of course, under the circumstances I had no option but to be bound by the written resolution of my colleagues whom I represented, which had just been read, and when the President asked me if I acquiesced in the rules, I referred to the resolution of my colleagues which lay on the table, while he appears to have referred to the resolution as he was about to propose it. But I certainly did not understand the drift of the President's question would be that if I acquiesced I should thereby make my colleagues acquiesce whether their assumptions were fulfilled or not. However, I at once pointed out that the Faculty did not contemplate that the rule including the term "any other professional appointment," would be feasible, as they stated in their resolution, and that they "assumed" that this would be subject to alteration, so as not to cover many appointments that might come under this term, and which they felt could never have been intended to be referred to under these terms. Amongst those I cited professorships and lectureships, appointments under the Midwives Act, appointments, for instance, at an eye hospital, examinerships in lunacy, appointments as visitors to asylums, and so forth, all of which would naturally be filled by experts in their own departments; and that therefore as we hoped some of these experts would be at the Royal Infirmary, it was *inconceivable* to the Faculty that the Committee should desire to exclude members of their own staff from taking such offices. I pointed out on behalf of the staff that anyhow the rule restricting the practice of physicians, surgeons and specialists to medicine, surgery and specialities respectively—to which they entirely assented, without reservation—was already going further than any other hospital, as far as rules were concerned, and would preclude their taking any other appointment that was not either purely medical, surgical or special; and that the exclusion of other appointments, such as those to which I have alluded, naturally held by physicians, surgeons, or specialists, and the exclusion of the ophthalmic surgeon at the Infirmary from holding appointment at any special eye hospital, would place many of their staff at a grave disadvantage.

I feel sure the omission to mention the remarks I made at the Committee must have been due to the fact that our esteemed President did not recall them at the time of speaking at the subsequent Board meeting, or else that the report of his remarks was not sufficiently full.

It is only fitting that I add that the Faculty as well as every one associated with the Bristol Royal Infirmary warmly appreciate the untiring devotion of their President to the progress and welfare of the charity which we all feel he has made his chief interest, though in this instance we are obliged to disagree as to the methods adopted by the Governors to attain the common aim.

